# Meta-analysis of the human gut microbiome uncovers shared and distinct microbial signatures between diseases

**DOI:** 10.1128/msystems.00295-24

**Published:** 2024-07-30

**Authors:** Dong-Min Jin, James T. Morton, Richard Bonneau

**Affiliations:** 1 Center for Genomics and Systems Biology, New York University, New York, New York, USA; 2 Center for Computational Biology, Flatiron Institute, Simons Foundation, New York, New York, USA; 3 Genentech, New York, New York, USA; University of California, San Diego, La Jolla, California, USA

**Keywords:** meta-analysis, microbiome, complex human diseases, disease similarity

## Abstract

Microbiome studies have revealed gut microbiota’s potential impact on complex diseases. However, many studies often focus on one disease per cohort. We developed a meta-analysis workflow for gut microbiome profiles and analyzed shotgun metagenomic data covering 11 diseases. Using interpretable machine learning and differential abundance analysis, our findings reinforce the generalization of binary classifiers for Crohn’s disease (CD) and colorectal cancer (CRC) to hold-out cohorts and highlight the key microbes driving these classifications. We identified high microbial similarity in disease pairs like CD vs ulcerative colitis (UC), CD vs CRC, Parkinson’s disease vs type 2 diabetes (T2D), and schizophrenia vs T2D. We also found strong inverse correlations in Alzheimer’s disease vs CD and UC. These findings, detected by our pipeline, provide valuable insights into these diseases.

**IMPORTANCE:**

Assessing disease similarity is an essential initial step preceding a disease-based approach for drug repositioning. Our study provides a modest first step in underscoring the potential of integrating microbiome insights into the disease similarity assessment. Recent microbiome research has predominantly focused on analyzing individual diseases to understand their unique characteristics, which by design excludes comorbidities in individuals. We analyzed shotgun metagenomic data from existing studies and identified previously unknown similarities between diseases. Our research represents a pioneering effort that utilizes both interpretable machine learning and differential abundance analysis to assess microbial similarity between diseases.

## INTRODUCTION

The progression of many complex human diseases has been validated to be influenced by the depletion of commensal microbes associated with health, as well as the presence of potentially pathogenic microbes. The intricate interplay between the commensal microbiota and the immune system has been shown to contribute to the pathogenic processes underlying various diseases (1, 2). In the context of standard microbiome studies, most diseases are typically studied in isolation. They are compared against a control group that is constructed to minimize confounding factors (3). This approach, however, neglects the fact that many patients have multiple comorbidities (4). Currently, nearly 60% of adult Americans have at least one chronic disease, with about 40% having multiple conditions (5). In medicine, it is common to study the nature of the comorbidities to understand the etiology of a given disorder. Within the field of oncology, genetic sequencing has been used to unravel similarities between disorders that were not observed from traditional medical observation (6). Doing so has provided a scaffold for drug repositioning to repurpose drugs to target similar cancer types.

We propose that the same approach should be taken when conducting microbiome studies. Existing literature also offers strong motivation for undertaking this study. First, microbes function as a central hub for the metabolism of dietary compounds, thereby serving as a coordination point for the distribution of nutrients (7). Consequently, it is plausible that any metabolic disorder may have an associated microbiome component (8). Second, microbes have a strong interaction with the immune system (9), thus play a critical role in the development and modulation of the immune system. Third, microbes are known to metabolize ingested drugs and contribute to efficacy (10, 11). All these facts indicate that microbes can have a multifaceted impact on disorders that have not been previously linked to the gut microbiome. Consequently, it is highly possible that there are disorders that are observed to have similar microbiome patterns, despite having dissimilar disease phenotypes.

Previous studies have shown that the imbalance of the bacterial community played a contributory role in the development of complex disorders, including neurological disorders, immune disorders, metabolic disorders, and gastrointestinal disorders. Recent studies have further highlighted the shared microbial signatures that contribute to these diseases, underscoring the need for more in-depth studies. For instance, *Prevotella copri* was found to be more prevalent in both type 2 diabetes (T2D) and rheumatoid arthritis patients compared to healthy controls, possibly due to its immune-relevant role in the pathogenesis (12, 13). More recently, dysregulation of the gut-brain axis has been demonstrated to contribute to the development of several neurological disorders, such as Alzheimer’s disease (AD), autism spectrum disorder (ASD), and mood disorders (14, 15). We explored the intersection of microbial signatures associated with those disorders, leveraging existing data sets that delve into the gut microbiome of these conditions.

Our goal is to provide a computational pipeline that can measure disease similarity based on microbiome composition. We utilize interpretable machine learning and differential abundance methods to identify both disease-specific microbes and microbes that are commonly observed across diseases. The key to comparing multiple disease cohorts was leveraging recent insights from removing batch effects within studies (15, 16). While previous meta-analysis research has mainly focused on analyzing multiple diseases to understand what makes each disease unique (17, 18), our pipeline represents the largest shotgun metagenomic meta-analysis conducted to measure the similarity between diseases with high resolution. We focus on diseases that are found to be associated with the imbalance of the gut microbiome. We included data sets investigating 11 disorders ranging from metabolic disorders, gastrointestinal (GI) disorders, and neurological disorders to cancer.

Since estimating disease similarity is a necessary first step prior to drug repositioning, we provide a modest first step in highlighting the possibility of incorporating microbiome insights into the drug-repositioning pipeline. To investigate the similarity between these disorders, we focus on shotgun metagenomics. While there are a lot more 16S rRNA gene data available, we have opted not to include these due to the lack of species resolution and genomic insights. This allows us to obtain high-level species or strain resolution while gaining insights into the potential functional roles of these microbes. We address a critical gap in understanding complex diseases by examining shared microbial signatures.

## RESULTS

**Fig 1:**
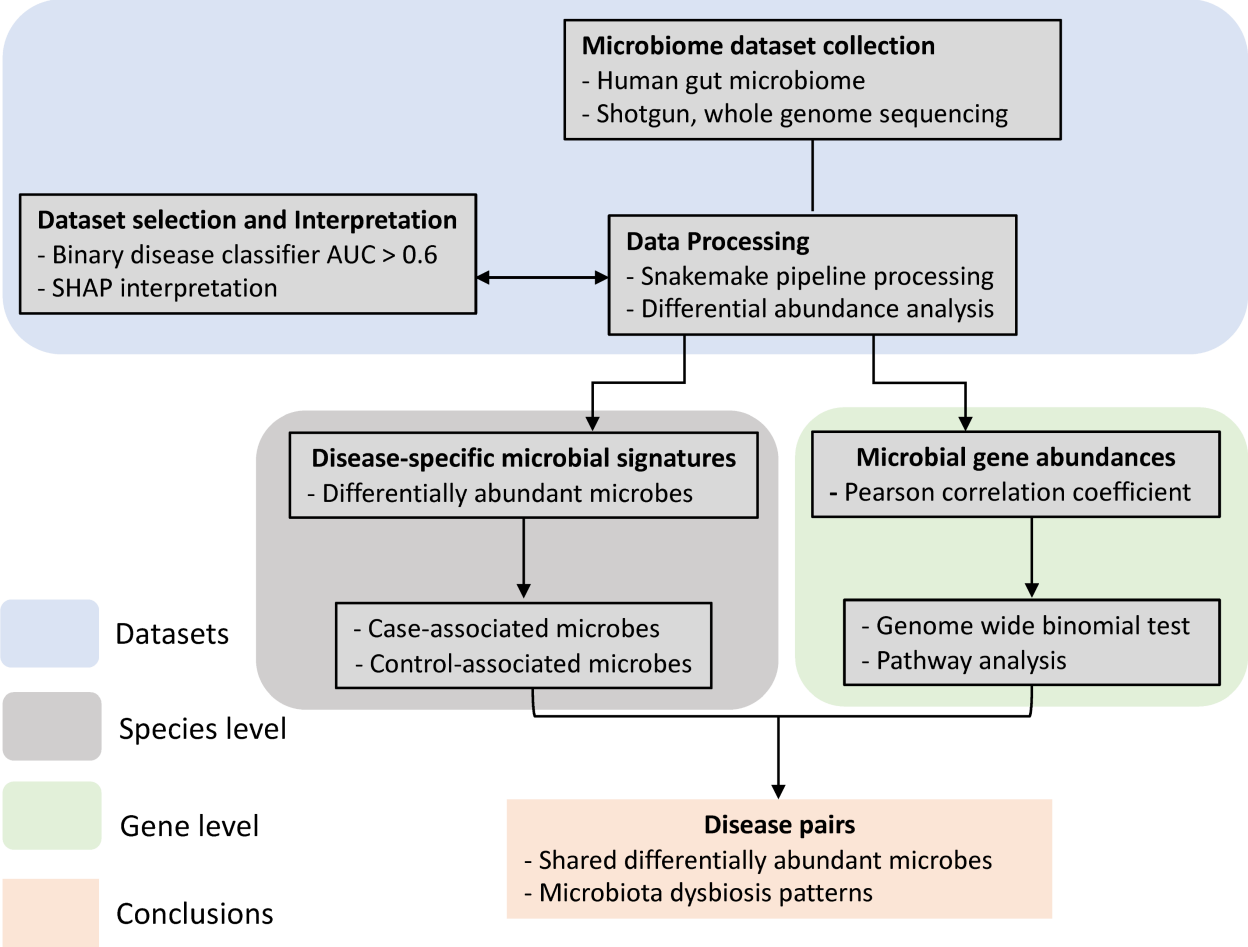
The overall design and data analysis pipeline. Flow chart of this meta-analysis. First, shotgun metagenomic data sets investigating the human gut microbiome in multiple diseases were curated and processed consistently with the Snakemake metagenomic pipeline built in this study, and the microbial abundances’ matrices were generated. Second, gradient boosting and random forest classifiers were built for each data set/disease. Then, data sets with classification accuracy above the threshold of 0.6 remained in the following analysis. Disease-specific microbial signatures and microbial similarity at the species level were analyzed with the differential abundance results. At the gene level, Pearson’s correlation coefficients of microbial genes between every disease pair were calculated and used as the proxy for disease similarity. Disease pairs that showed high or low similarity were further investigated with pathway analysis.

We have developed a novel pipeline (Fig. 1) that computes disease similarity at both microbial species and gene level, enabling a consistent data standard to make different studies more comparable. We compiled a large multi-study meta-analysis with consistent processing to enable comparisons across studies that account for batch effects. Our findings reveal a high degree of similarity between Crohn’s disease (CD) vs ulcerative colitis (UC), CD vs colorectal cancer (CRC), Parkinson’s disease (PD) vs T2D, as well as schizophrenia vs T2D. Our results show that the similarity at the microbial-species level was consistent with the similarity at the microbial-gene level, explained by both the enrichment of pathogenic microbes and the depletion of beneficial microbes. Finally, we found that the microbial gene profiles between AD and inflammatory bowel disease (IBD) are anticorrelated, highlighting a more pronounced metabolic distinction between these two disorders than previously suspected.

### Consistent data processing and cohort selection for this meta-analysis

In this study, we applied the Snakemake pipeline to process available samples and constructed binary disease classifiers for each study. After fitting both binary gradient boosting (GB) and random forest (RF) classifiers, we found that GB classifiers showed better overall performance across diseases. We then employed GB classifiers in subsequent studies and utilized them to exclude studies that cannot discriminate the disease phenotype based on microbial profile. The resulting data set derived from 18 studies (19–35), encompasses a total of 2,091 samples (Table 1). A summary of the sample preparation and sequencing information has been provided in Table 2. Most studies stored their sample in −20°C for a short term before transferring to the −80°C refrigerator. Although these studies used different DNA extraction kits and sequencing platforms to generate the sequencing data, the strategies employed were consistent within each study. When computing the abundances of species in DNA sequences, read lengths are specified based on the characteristics of the sequencing data per study/batch in bracken with “-l ${READ_LEN}.” These samples are distributed across 11 countries spanning Europe, Asia, and America. The following diseases were included: four neurological disorders (AD, ASD, schizophrenia, and PD); two autoimmune disorders (multiple sclerosis [MS] and type 1 diabetes [T1D]); two metabolic disorders (obesity and T2D); two GI disorders (CD and UC); and one cancerous disorder (CRC). We compared the classifier performances per cohort and per disease. Per cohort refers to build classifiers for each data set, and per disease refers to build classifiers with the data sets for a disease combined. The results showed that the overall classification accuracy increased when it was tested per disease, suggesting that the consolidation of data sets from diverse cohorts enhances the overall representativeness of the disease, consistent with previous findings investigating both ASD (15) and CRC (26).

**TABLE 1:** Disease and metagenomic data sets included in this meta-analysis^*a*^

Disease	Cases (*n*)	Controls (*n*)	RF AUC	GB AUC	Age (year)	Country	Reference
AD	75	75	0.49	0.66	49–82	Germany	(19)
ASD	30	30	0.85	0.65	3–13	China	(20)
ASD	31	31	1	1	2–6	China	(36)
ASD	64	64	0.68	0.62	3–6	China	(22)
CD	54	54	1	0.9	21–82	The Netherlands and USA	(23)
CD	52	52	0.95	0.97	13–51	China	-
CRC	40	40	0.99	0.8	43–86	Austria	(24)
CRC	52	52	0.82	0.9	24–87	France	(25)
CRC	49	49	0.99	0.99	28–90	Germany	(26)
MS	105	105	0.5	0.65	18–74	UK and USA	(37)
Obesity	45	–	0.65	0.63	–	Denmark	(28)
Obesity	36	36	0.57	0.8	42–69	Denmark	(29)
PD	88	88	0.82	0.82	37–84	USA	(30)
PD	40	40	0.87	0.98	58–74	China	(31)
Schizophrenia	81	81	0.75	0.71	17–64	China	(32)
T1D	53	53	0.97	0.88	1–3	Finland and Estonia	(33)
T2D	76	76	0.77	0.77	14–78	China	(29)
UC	59	59	0.7	0.87	18–68	Spain	(35)
UC	76	–^*b*^	–	–	19–80	USA and the Netherlands	(23)

^*a*^ AUC, area under the receiver operating characteristic curve.

^*b*^ “–” indicates data points that are not applicable/available for the given category or metric.

**TABLE 2:** Summary of sample preparation for the included studies

Study	Disease	Sample storage	DNA extraction	Sequencing platform	Read length (bp)
(19)	AD	−20°C	Zymo Kit D4300	Illumina HiSeq 2000	150
(20)	ASD	−80°C	Tiangen Kit DP328	Illumina HiSeq X Ten	150
(36)	ASD	−80°C	StoolGen DNA Kit	Illumina HiSeq 4000	150
(22)	ASD	–^*a*^	Promega Kit	Illumina NovaSeq 6000	150
(23)	CD, UC	−80°C	QIAGEN Kit	Illumina HiSeq 2500	100
CD_Chinese	CD	–	–	Illumina HiSeq 2000	100
(24)	CRC	−80°C	–	Illumina HiSeq 2000	100
(25)	CRC	−80°C	GNOME Kit	Illumina HiSeq 2000	100
(26)	CRC	–	–	Illumina HiSeq	150
(37)	MS	−80°C	QIAGEN Kit	Illumina HiSeq 4000	150
(28)	Obesity	−80°C	–	Illumina Genome Analyzer II	44–90
(29)	Obesity	−80°C	–	Illumina Genome Analyzer II	88–150
(30)	PD	−20°C	QIAGEN Kit	Illumina NovaSeq 6000	150
(31)	PD	−80°C	QIAGEN Kit	Illumina HiSeq X Ten	150
(32)	Schizophrenia	−80°C	–	BGISEQ-500	150
(33)	T1D	−80°C	–	Illumina HiSeq 2000	100
(29)	T2D	–	–	Illumina Genome Analyzer II	100
(35)	UC	–	–	Illumina Genome Analyzer II	77

^*a*^ “–” indicates data points that are not available for the given category.

Our analysis revealed three diseases with within-cohort cross-validation area under the receiver operating characteristic curve (AUC) exceeding 0.95 utilizing different machine learning algorithms in certain data sets: ASD, CD, and CRC (Table 1). Notably, both CD and CRC are diseases related to GI, predominantly impacting the GI tract. Within the ASD cohort, where we observed high classification accuracy, most of the ASD patients also have GI symptoms (36). The classifier is trained on the training data set, and its predictive accuracy is assessed on a hold-out test data set. This is important to emulate real-world clinical environments, where there could be a drift between clinical studies due to confounding demographics and experimental protocols. Additionally, we collected a hold-out cohort evaluation using two independent data sets to assess the generalization performance of the binary classifiers on previously unseen test data sets for CD (35) and CRC (38).

### Comparison of Crohn’s disease and colorectal cancer: SHAP interpretation and differential abundance analysis

As population-based cohort studies have found that CD is a risk factor for CRC (39), we chose to compare the shared microbial signature between CD and CRC first as a sanity check for our analysis. We applied two distinct approaches to gain insights. First, we used Shapley additive explanations (SHAP) to interpret the binary disease classifiers. SHAP provides a valuable means of understanding the contribution of each feature in the classification process, offering interpretability to complex machine learning models. Second, we conducted a comprehensive analysis of differential abundance. This approach allows us to identify significant variations in the abundance of microbes between disease cases and healthy controls. Since these two quantities are generated from distinct methods, they provide different perspectives that are sometimes in conflict. By leveraging both pieces of information, we looked at microbes that could be strongly explained by both the Shapley values and large log fold changes. While we did not observe an overlap in the features that contribute most to the classification for CD and CRC, we found there is considerable overlap in the microbial species that exhibit differential abundance in CD and CRC patients.

**Fig 2:**
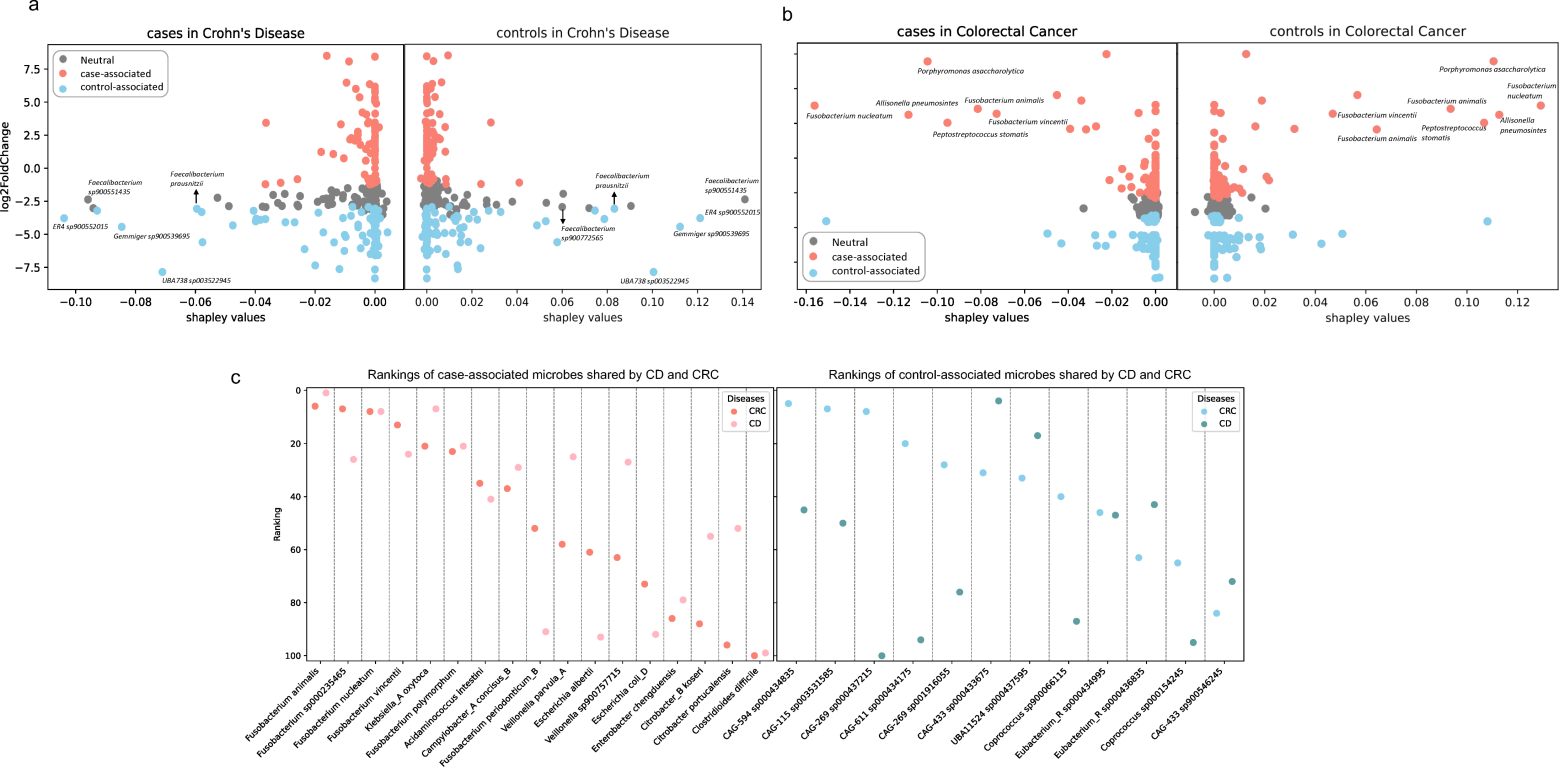
Interpretation of binary classifiers and differentially abundant microbes’ overlaps in CD and CRC. (a) Shapley values vs log2 fold change (LFC) in CD cases and controls. *x* axis is the Shapley values, and *y* axis is the log2 fold change between case and control. Left panels are the cases, which have a sum of Shapley values as negative values, right panels are the controls, which have a sum of Shapley values as positive values. The differentially abundant microbes are identified first by computing LFC between case and control within one disease, then ranked by the 5% confidence interval (CI) of LFC to identify the top 100 case-associated microbes, and finally ranked by the 95% CI of LFC to identify the top 100 control-associated microbes. Each dot represents one microbe, and its color is coded by its ranking. Dots colored blue and salmon represent the microbes differentially abundant in disease cases and controls, respectively. Dots colored gray are the ones that are considered neutral. Dots with high absolute Shapley values and high LFC are labeled. (b) Shapley values vs LFC in CRC cases and controls. Same representation as shown in panel a, but for CRC. (c) Overlap of the differentially abundant microbes between CD and CRC. *x* axis is the microbes, and *y* axis is the microbe’s rankings. A smaller ranking number for case-associated microbes indicates a greater increase of the microbe in disease cases. A smaller ranking number for control-associated microbes indicates a greater increase of the microbe in healthy controls.

Both the binary classifiers of CD and CRC displayed robust generalization abilities when tested on previously unseen cohorts, with AUC values of 1.00 and 0.87, respectively. In the case of CD classification, control-associated species within the *Faecalibacterium* genus, such as *Faecalibacterium sp3900551435* (Shapley rank first in CD control and second in CD case) and *Faecalibacterium prausnitzii* (Shapley rank sixth in CD control and seventh in CD case), exhibit high absolute Shapley values in both cases and controls of the CD cohort (Table S1). As demonstrated in prior research, *F. prausnitzii* can produce proteins with anti-inflammatory properties and is involved in CD pathogenesis (40). Altogether, our results imply that these control-associated microbes played a substantial role in distinguishing CD patients from controls (Fig. 2a).

However, in CRC, case-associated microbes had a more pronounced influence on CRC classification (Fig. 2b), particularly exemplified by *Fusobacterium nucleatum*, *Allisonella pneumosintes*, and *Prophyromonas asaccharolytica* (Table S2). Specifically, *F. nucleatum* was ranked first in terms of Shapley values in both CRC case and control groups. *F. nucleatum* was known to be enriched in colorectal adenomas and adenocarcinomas (41), and it can create a proinflammatory microenvironment that supports the progression of colorectal neoplasia (42). It is also one of the common oral bacteria. This has been previously observed in lung cancer, where oral commensals are more abundant in the lower airway of lung cancer patients compared to the control population (43). Recent studies found the connection between oral bacteria and gut is possibly through both ectopic gut colonization by oral commensals and induction of migratory Th17 cells, constituting a complex interplay between the microbiome and immune system (44, 45). Our results confirmed its pivotal role in distinguishing CRC patients from controls. We also identified other candidates that warrant further investigation.

**Fig 3:**
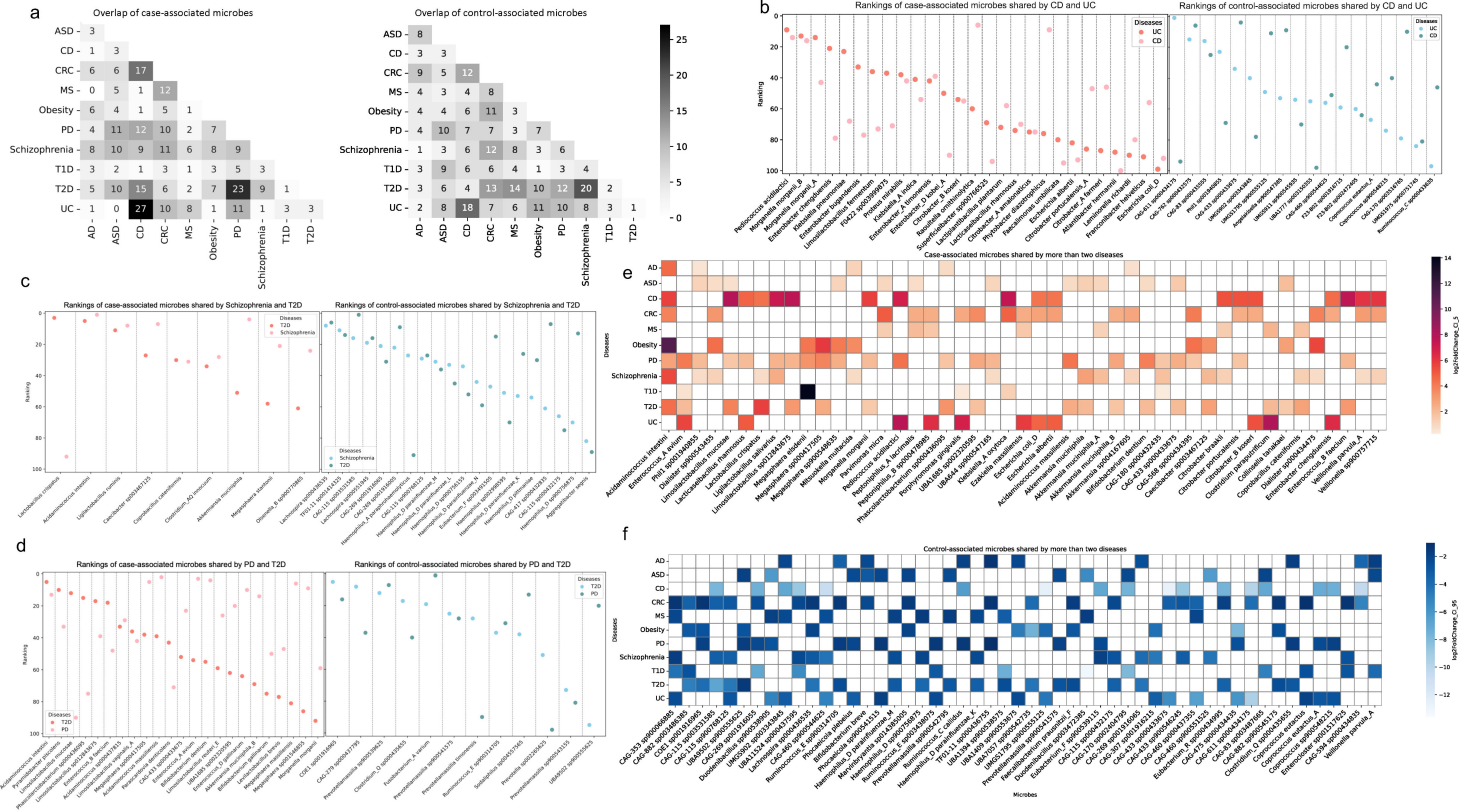
Microbial species-level similarity between diseases. (a) Overlap of case-associated and control-associated microbes. The annotation numbers represent the number of microbes overlapping between two diseases among the top 100 case-associated microbes or the top 100 control-associated microbes. (b) Overlap of the differentially abundant microbes between Crohn’s disease and ulcerative colitis. Dots colored in salmon represent case-associated microbes and their rankings. A smaller ranking number indicates a greater increase of the microbe in disease cases. Dots colored in blue represent control-associated microbes and their rankings in controls. A smaller ranking number indicates a greater increase of the microbe in healthy controls. (c) Overlap of the differentially abundant microbes between schizophrenia and T2D. (d) Overlap of the differentially abundant microbes between PD and T2D. (e) Case-associated microbes shared by more than two diseases. *x* axis is the microbes, and *y* axis is the diseases, colored by the LFC values between case and control within each disease. (f) Control-associated microbes shared by more than two diseases. Same representation as shown in panel e, but for control-associated microbes.

It has been found that individuals diagnosed with CD may face an elevated risk of developing CRC, possibly due to the chronic inflammation associated with CD (39, 46). Our findings revealed the overlapped case-associated microbes between CD and CRC contributed to the similarities of these two diseases, such as *Fusobacterium* spp. and *Veillonella* spp. along with the shared depletion of potential probiotic *Coprococcus* spp. (Fig. 2c). Both diseases showed an increase in key components of the human gut microbe such as *Escherichia coli*, with mean relative abundance of 0.059 and 0.033 in CD cases and CRC cases, respectively (Fig. S1a and b). It is worth noting that we also found many *Fusobacterium* species worth looking into (*Fusobacterium animalis, Fusobacterium sp000235465, Fusobacterium nucleatum, Fusobacterium vincentii,* and *Fusobacterium polymorphum*), including one of them (*F. prausnitzii*) that has been validated by previous studies (40). Differential abundance analysis found 19% and 17% overlap of the case-associated and control-associated microbes, respectively, between these two diseases (Fig. 3a). In the original studies that generated the CRC data sets, *Fusobacterium* spp. were identified as one of the most significant markers for CRC patients by Wirbel et al. (26), and *Veillonella* spp. were identified as CRC-enriched microbes by Feng et al. (24). On the other hand, in the original study that generated one of the CD data sets, Franzosa et al. (23) found that *Coprococcus* spp. were correlated with non-IBD controls. Our findings, in agreement with previous studies, highlight the potential involvement of these shared microbial signatures in the progression of both CD and CRC. The shared microbial features we find here are crucial for us to better understand the common features between these diseases and can help us step closer to the real therapeutic target for these complex diseases.

### Differential abundance analysis revealed disease pairs with high similarity at the microbial species level

Among the top disease pairs exhibiting the most significant overlap of case-associated microbes, CD vs UC had the highest co-occurrence, followed by PD vs T2D (Fig. 3a). For case-associated microbes, 27% were common to both CD and UC, and 23% were shared between PD and T2D. On the other hand, schizophrenia vs T2D, as well as CD vs UC, showed a substantial overlap of control-associated microbes. Specifically, 20% and 18% of the top control-associated microbes were shared in these pairs, respectively (Fig. 3a). We chose to conduct a more in-depth comparison at the microbial species level for those disease pairs: CD vs UC, PD vs T2D, and schizophrenia vs T2D. Based on the observation that these pairs exhibited the highest overlaps of differentially abundant microbes, we identified the shared differentially abundant microbes for these three disease pairs. Differentially abundant microbes shared by more than two diseases have also been identified in our study (Fig. 3).

Among the case-associated microbes shared by CD and UC, recognizable microbes, such as *Pediococcus acidilactici* and known commensal species like *Morganella morganii* (Fig. 3b and e), are potentially involved in gut dysbiosis. A recent study in mice has confirmed that the overabundance of *P. acidilactici* may play a role in triggering IBD by producing lipopolysaccharide and exopolysaccharide byproducts (47). Cao et al. (48) have demonstrated that *M. morganii* isolated from IBD patients can generate genotoxic metabolites called indolimines. These metabolites have the potential to induce DNA damage and contribute to cancer progression. Within the control-associated microbes, *Coprococcus eutactus*, a potent probiotic that can alleviate colitis through acetate-mediated IgA response and microbiota restoration (49), is among the most prevalent in the control populations. Our results unveiled several other case-associated microbes that warrant further investigation, including species from the genera *Enterobacter* and *Citrobacter*, among others (Fig. 3b). Altogether, this highlights the strong microbial similarity between the two IBD subtypes, which is consistent with both previous microbiome studies and the clinical phenotype (23, 50).

We found that control-associated microbes depleted in both schizophrenia and T2D including species from the *Lachnospira* and *Haemophilus* genera (Fig. 3c and f). Microbes from the Lachnospiraceae family are known to produce butyrate, which is one of several SCFAs that has beneficial effects on cellular metabolism and intestinal homeostasis. The loss of such microbes is linked to chronic inflammation and is likely involved in metabolic diseases such as T2D (51). On the other hand, *Haemophilus parainfluenzae* is a common commensal that has been recognized as an opportunistic pathogen, but its specific functional role remains unclear. Studies have reported a lower abundance of *H. parainfluenzae* in mental disorders compared to healthy controls (52). We found the decreased abundances of these microbes contribute to the similarity between schizophrenia and T2D.

Similar to schizophrenia, PD is another neurological disorder that showed high similarity with T2D, however, mainly contributed by the shared increase of case-associated microbes in patients (Fig. 3d). We observed that *M. morganii* is also differentially increased in both PD and T2D patients, along with species from the genera *Acidaminococcus*, *Limosilactobacillus*, and others. The increased abundance of *Acidaminococcus intestini* in disease cases has been found in seven diseases (Fig. 3e), making it the most commonly shared case-associated microbe in our analysis. A recent cross-sectional study found *Acidaminococcus intestini* was one of the microbes that were more abundant in subjects consuming the most pro-inflammatory diets (53). Consistent with the observations in the original studies that generated these data sets (31, 32, 34), we did observe that *Akkermansia muciniphila* was differentially increased in all these disorders (PD, schizophrenia, and T2D) as well (Fig. 3e). In the study that generated the other PD cohort, Wallen and colleagues (30) found that *Akkermansia* abundances might be affected by the geographic locations. They were able to identify this signal in a prior multi-state 16S data set that was primarily from the northern US but not the data set from the southern US. However, the underlying mechanisms are not clear. This finding is highly controversial in the literature since previous studies have observed that *Akkermansia muciniphila* is both beneficial (54) and pathogenic (37). From our analysis, it is difficult to determine the causal role of *Akkermansia muciniphila* in these diseases. Follow-up mechanistic and clinical studies will be necessary to explore the involvement of this microbe in more depth.

### Microbial gene-level comparison and pathway analysis showed consistency with species-level results

**Fig 4:**
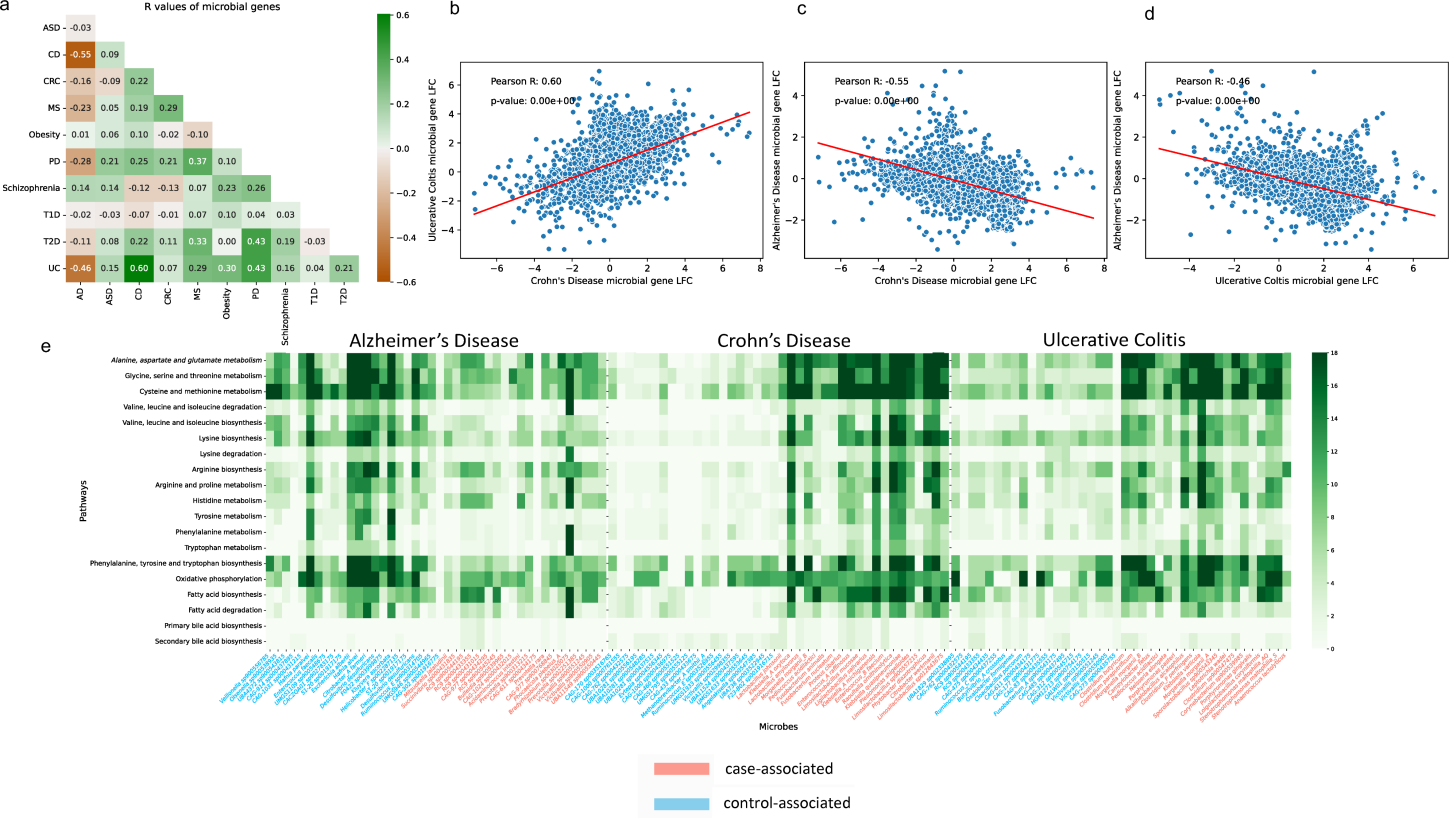
Microbial gene-level similarity between diseases and the pathway signatures of the microbes. (a) Pearson’s correlation coefficient *R* between the inferred microbial gene log2 fold changes across every two diseases. (b) Scatterplot of the Pearson *R* between Crohn’s disease and ulcerative colitis. (c) Scatterplot of the Pearson *R* between CD and AD. (d) Scatterplot of the Pearson *R* between UC and AD. (e) Amino acid metabolism, energy metabolism, and lipid metabolism pathways of the microbial signatures in AD, CD, and UC. The *x* axis is the differentially abundant microbes, the blue ones represent control-associated microbes, while the salmon ones represent the case associated. The *y* axis is the KEGG pathway module. The numbers on the right green bar represent the number of genes.

Disease similarity based on the microbial genes can be accessed by comparing the Pearson’s correlation coefficient *R* between the inferred log2 fold changes (LFC) across every two diseases (Fig. 4a), *P*-value has been included in Table S3. The *R* value for CD vs UC stands at 0.6, representing the highest positive correlation observed across all disease pairs (Fig. 4b). As two subtypes of IBD, both CD and UC are characterized by transmural inflammation, with CD being able to affect any area from the mouth to the perianal region, while UC is limited to the colon’s mucosal layer (55). Previous studies have demonstrated that IBD is influenced by genetic predisposition, immune system dysregulation, and environmental factors (23, 35). Our pathway analysis revealed the involvement of both case-associated and control-associated microbes in various metabolic pathways. Specifically, we identified discrepancies in amino acid metabolism, energy metabolism, and lipid metabolism that differentiated between case-associated microbes and control-associated microbes for CD and UC. Case-associated microbial genes exhibited a higher prevalence within most of these metabolic pathways, many of which contributed to inflammation and infection (Fig. 4e). Pathogenic microbes such as *Fusobacterium*, *Klebsiella,* and *Stenotrophomonas* are heavily involved in pathways including tryptophan biosynthesis, oxidative phosphorylation, and fatty acid biosynthesis. This is consistent with findings from previous studies (56–58), highlighting the microbial and clinical similarity between these two disorders.

Conversely, AD has a strong negative correlation between differential gene abundances between both CD (*R* = −0.55) (Fig. 4c) and UC (*R* = −0.46) (Fig. 4d), highlighting how the microbial link with AD affects the same pathways, but possibly through a different mechanism of action. Among the genes that were most differentiating between cases and controls for CD, UC, and AD, many of these genes are involved in the pathways related to the metabolism of amino acid, lipid, and energy. In AD patients, this was partially due to the decrease in microbes, such as the ones from the genera *Veillonella, Hafnia*, *Ruminococcus*, and *Citrobacter*, which had a greater prevalence of genes that are encoded in these pathways (Fig. 4e). Some of the control-associated microbes are known to generate metabolites like histamine, conjugated fatty acids, and dopamine, which act as neuroprotective agents in AD (59). AD is known for the accumulation of beta-amyloid plaques and tau tangles in the brain (19) and is often characterized by metabolic abnormalities, including compromised bioenergetics, impaired lipid metabolism, and an overall decreased metabolic capacity (60).

Most of the drugs designed to treat AD patients, such as lecanemab, donanemab, and remternetug, are focused on removing plaque (61). In contrast, many drugs that target IBD are immunosuppressants, such as azathioprine, mercaptopurine (6-MP), and methotrexate (62). While drugs used to treat IBD and AD are known to have very different functional roles, it is interesting to see how the microbial gene profiles between these two disease populations have discordance in the same metabolic pathways (Fig. 4e). It is currently not clear to us why this discordance exists. However, these findings highlight interesting directions for pre-clinical follow-up studies, particularly in exploring the utility of immune-enhancing drugs in AD.

### Both the enrichment of pathogens and depletion of control-associated microbes contribute to the similarity between complex human diseases

Various types of microbiome shifts in complex human diseases have been identified by previous studies, encompassing the depletion of beneficial microbes, enrichment of pathogens, and a comprehensive reconstruction of gut microbial communities (17). In many of the disease pairs that exhibit a high overlap in microbial signatures, we found that both the enrichment of pathogens and the depletion of beneficial microbes contribute substantially to their similarity. This holds true regardless of the previous classification of their dysbiosis patterns in prior studies.

**Fig 5:**
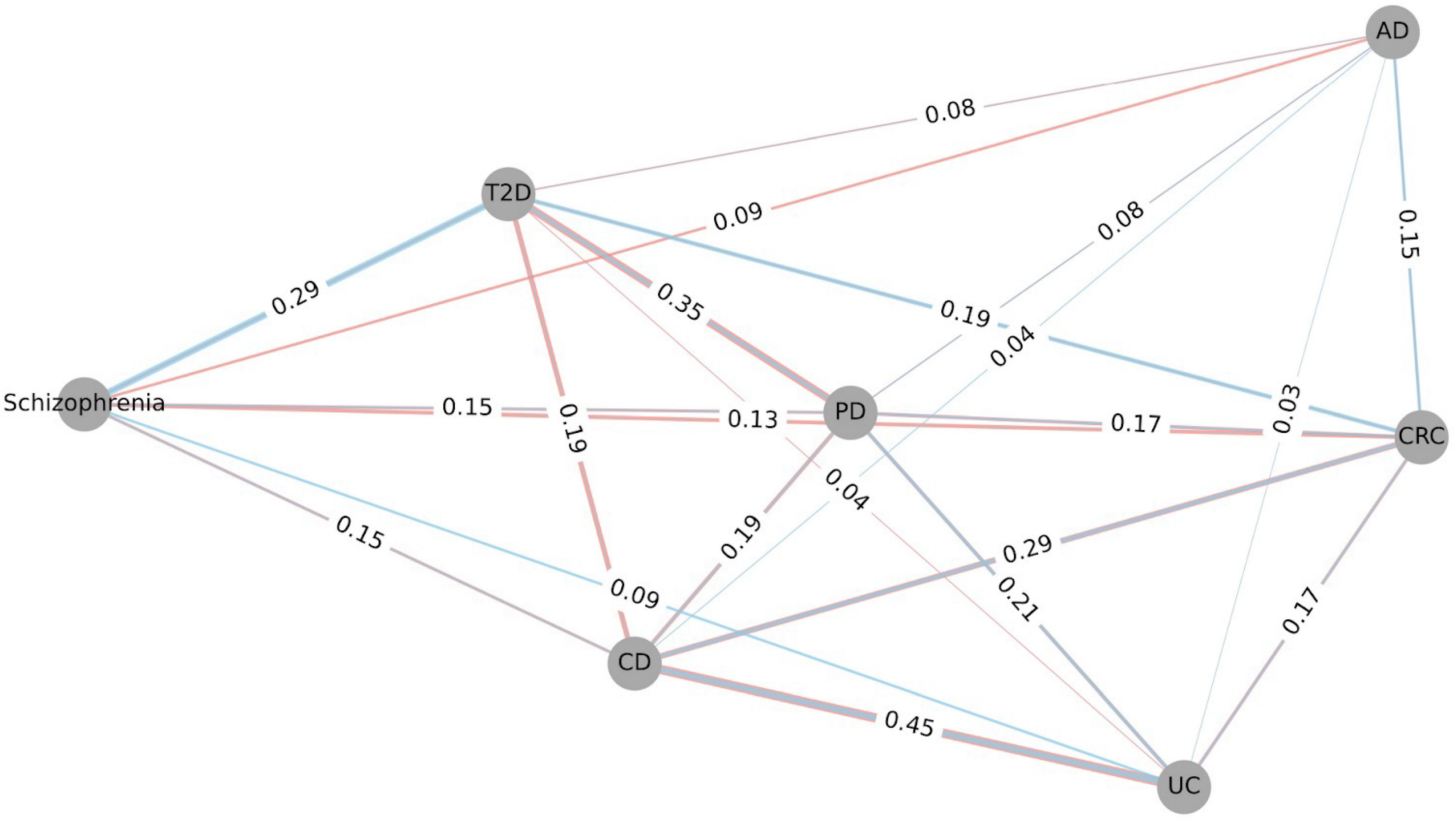
Combined similarity networks with the sum of overlapped microbe weights. Each node represents one disease type, and the weight of edges shows how similar the two diseases are. The number in each edge is proportional to the overlapped differentially abundant microbes in each disease (case vs control): top 100 (case associated) and bottom 100 (control associated). The colors of the edges indicate the origin of the similarities: salmon color edges represent the similarity conferred by the overlap of case-associated microbes; the blue color represents the similarity conferred by the overlap of control-associated microbes.

Dysbiosis associated with CRC was generally characterized by increased prevalence of the pathogenic microbes (25), while CD was consistently characterized by the depletion of control-associated microbes (63). Combined similarity networks with the sum of overlapped microbe weights show that both shifts contribute to the similarities between diseases (Fig. 5). The color of the edges shows the difference in shifts, and the width of edges between two diseases is proportional to the overlapped microbes. The similarities between CD and CRC comprise a mixture of both shifts. This indicates that the dysbiosis patterns of some diseases are more complicated than initially clarified, opening new opportunities for repurposing narrow-spectrum antimicrobials and probiotic treatments.

There is consistency between the similarity observed at the microbial species level and that at the microbial gene level. AD and the two IBD subtypes showed the least overlap in differentially abundant microbes. They also exhibited the least similarities at the microbial gene level. Furthermore, disease pairs like CD vs UC, CD vs CRC, PD vs T2D, as well as schizophrenia vs T2D demonstrated a high overlap in differentially abundant microbes and high Rs in microbial gene abundances. Discrepancies may arise when comparing these similarities at different levels. For instance, both PD vs UC and PD vs T2D have strong microbial gene similarities (*R* = 0.43 and *R* = 0.43) (Fig. 3a). However, PD vs UC has a very small overlap in differentially abundant microbes (overlap = 21%), while PD vs T2D has a larger overlap (overlap = 35%) (Fig. 5). This is consistent with the functional redundancies that have been observed in microbial communities; even if there is a small overlap between the microbial taxa, there could still be a strong overlap in the metabolic function due to the common metabolic roles different microbes play (64).

## DISCUSSION

We have assembled the largest shotgun metagenomics meta-analysis that has inferred disease similarity with high resolution, across 2,091 samples from 18 studies, encompassing 11 different disease types. We conducted a case-control differential abundance analysis within each disease, following a comparison between diseases. Our results demonstrated that binary disease classifiers for CD and CRC exhibit a strong generalization capability when applied to unseen data. We discovered a high degree of microbial similarity between CD and CRC. This finding aligns with the fact that CD is a risk factor for CRC, thereby validating our pipeline. Furthermore, CD and UC are detected to have the strongest microbial similarity. Given that both are subtypes of IBD, this observation further substantiates the effectiveness of our pipeline.

We identified two neurological disorders, PD and schizophrenia, which exhibited high microbial similarity with T2D. The T2D cohort included here is a group of individuals who had not received any antibiotic treatment within 2 months before sample collection (34). The schizophrenia cohort only contains treatment-naive patient recruitment by Zhu et al. (32). In the two PD cohorts included here, while the treatment for the American cohort is not clear (30), the Chinese cohort has excluded patients with antibiotic use within 3 months prior to sample collection (31). The Chinese PD patients had additional medication usage, including levodopa, dopamine agonists, and several other anti-parkinsonism drugs. We did not exclude the effects of drug treatment to our identified signatures, especially for the PD cohorts, as they cannot be adjusted due to the limited available information.

The higher prevalence of T2D in schizophrenia patients has been observed in observational clinical studies (65), but there has not been a microbiome connection that has been previously established. Our findings offer valuable perspective on the potential for repositioning T2D drugs to treat these neurological disorders or vice versa. Metformin is a commonly used oral treatment for T2D (66). Metformin alters the gut microbiome of T2D patients, and altered gut microbiota mediates some of metformin’s antidiabetic effects (67). Interestingly, recent studies suggest that metformin has a positive effect on conditions such as anxiety or depression (68, 69). A mouse study also established the neuroprotective effect of metformin in PD and supported the therapeutic potential of metformin in the treatment of PD (70, 71).

One surprising finding we discovered was that microbiome profiles in AD were anti-correlated with microbiome profiles in IBD. Microbiome components have been observed to play a role in both diseases. In a study on AD involving fecal microbiota transplantation (FMT), fecal microbiota from Alzheimer’s patients and age-matched healthy controls were transplanted into microbiota-depleted rats, respectively. It was observed that the severity of impairments in hippocampal neurogenesis in these rats correlated with the clinical cognitive scores of the donor patients (72). Multiple FMT in IBD have shown promising results in reducing inflammation in patients (73, 74). However, common biological mechanisms between these two diseases have not been previously established. AD is characterized by inflammation in the brain, while IBD is mainly characterized by GI inflammation. In the original study that generated the AD data set, Laske et al. (19) built a mode for discriminating between AD patients and healthy controls. *Veillonella* was one of the genera that was included in their model and was found to have higher abundance levels in controls. We confirmed that *Veillonella* spp. were control-associated in our analysis of the AD cohort. However, *Veillonella* spp. were found to be case-associated in the CD cohorts in our analysis and also in other studies (63, 75). One possible explanation for this could be the genomic diversity differences of these microbes contributed to the microbial dysbiosis pattern differences. The anti-correlated microbial gene profiles between AD and IBD also highlight potentially novel directions for drug design. If drugs designed to target IBD were applied to Alzheimer’s patients, would they antagonize Alzheimer’s symptoms? Furthermore, is it possible for these efforts to uncover new therapeutic strategies that could counteract the effects of these drugs?

While our findings provide valuable insights, it is important to note that our study is subject to several notable limitations. First, there are multiple confounding factors that could bias our findings. For instance, most studies did not perform absolute quantification, thus it is not possible to identify microbes that are truly differential between the case and control populations (76). It is possible that the microbes detected were due to our choice of reference frame, we assume that the average microbe is not changed between the case-control cohorts, but if there is a significantly altered microbial load between the cohorts, that could lead to false positives or false negatives in the differential abundance results (76). To ameliorate this issue, we focused on the top 100 microbes differentially increased in the cases and the top 100 microbes differentially increased in the controls to avoid the issue of identifying an unstable reference frame. There are also likely few biological confounders that are not well-documented but could affect our findings, such as medication history (i.e., antibiotics usage) or dietary patterns.

To improve our ability to perform causal inference, it is important to not only account for these relevant confounders but also take advantage of longitudinal observational cohorts and clinical trials to identify indirect effects on outcomes due to external interventions. Incorporating multiple omics levels will also help improve causal resolution since increasing the number of observed biomarkers will increase the chances of observing a biomarker that plays a causal role in the disease symptoms. Our analysis focused solely on shotgun metagenomics data, overlooking the potential insights offered by other omics-level data (77). Host transcriptomic profiles facilitated the identification of host gene-microbiome associations in gastrointestinal disorders (78). Metabolomics would yield insights into lipid and bile-acid metabolism, which has been observed in the context of IBD (79). Proteomics will likely play an important role in understanding amino acid metabolism and immune response, which we have shown to play a role in estimating disease similarity. While observational and clinical studies can help identify putative causal biomarkers, preclinical studies with follow-up mechanistic experimentation are needed to confirm the causal roles of these biomarkers.

Furthermore, the availability of microbiome data sets presents obstacles to performing a more comprehensive microbiome-centric disease meta-analysis. Some diseases, such as T1D, have fewer microbiome studies, especially when compared to other conditions like IBD and CRC. Furthermore, most of the studies that we analyzed focused on a single disease, which by design excludes other disease comorbidities. At this moment, we are not aware of observational studies that investigate population-level comorbidities from a microbiome perspective. Our findings strongly suggest that broadening the range of microbiome data collection could significantly enhance the analysis of disease comorbidity. This would not only improve our understanding of known microbiome-associated diseases but could also unveil microbiome associations for disorders that have not been previously shown to have a microbiome component.

## MATERIALS AND METHODS

### Curate shotgun metagenomic data sets

The Sequence Read Archive (SRA) stands as the most extensive publicly accessible repository of sequencing data across various sequencing platforms. To identify studies and data sets exploring the human gut microbiome in the context of complex human diseases, we utilized the SRAdb package (https://github.com/seandavi/SRAdb). Using keywords such as “gut microbiome,” “human,” and “shotgun,” we identified relevant studies and data sets within the SRA repository. Data sets that have metadata available were selected and subjected to case-control matching within studies based on age and gender information. Samples were filtered to exclude individuals with obesity when BMI information was available. Unmatched samples were subsequently removed from the analysis. The retained samples then underwent consistent processing methods to generate microbial abundances. To streamline and automate the workflow, a Snakemake (80) pipeline was developed for this study, which can be accessed at https://github.com/jindongmin/snakemake_metagenomics. The pipeline takes the SRA BioProject IDs as input and outputs the microbial abundance biom tables. The workflow began with downloading the sequencing data with fasterq, which was followed by quality profiling and filtering steps with fastp (81). Kraken2 (82) and bracken (83) were employed to classify the reads to the best matching location in the taxonomic tree and compute the abundance of species.

In terms of Kraken2 databases, we benchmarked Web of Life and the Unified Human GI Genome version 2 (UHGG v2.0) databases (84). Our finding indicated that UHGG v2.0 offered a more comprehensive coverage of species at the time of our access to the databases. Consequently, we opted to utilize UHGG v2.0 in our study.

### Build disease classifiers and filter data sets

Machine learning algorithms including GB and RF were employed for cross-validation to assess the accuracy of gut microbes in distinguishing between disease cases and control subjects. We fitted classifiers for each data set using q2-sample-classifier (85), and each microbial abundance was treated as a feature. Binary disease classifiers for CD and CRC were constructed by combining all the data sets per disease. The samples were randomly divided into training and testing sets, with an 80/20 split. The training set was utilized to construct the model and obtain optimal model parameters, while the hold-out testing data set was used to generate predictions. The performance of GB and RF classifiers was evaluated across the data sets using the AUC. An AUC value of 0.5 indicates that the corresponding classification has the same predictive ability as random guessing. To ensure that the included data sets possessed discernible microbial signatures capable of distinguishing between cases and control subjects, we applied a threshold for an AUC of 0.6 and retained only those data sets with an AUC greater than 0.6.

### SHAP interpretation of binary disease classifiers

SHAP is an explanatory approach rooted in game theory that aims to shed light on the outcomes produced by machine learning models (86). It leverages Shapley values, which are a solution concept derived from cooperative game theory (87). These values provide insights into the individual contributions of players within a coalition game. In the context of SHAP, each microbe (represented by abundance) is considered a player (feature), and by calculating Shapley values, we can understand their respective influences on the predictions made by disease classifiers. Shapley values are calculated by considering all possible combinations (coalitions) of the microbes and evaluating the marginal contribution of each microbe to the prediction outcome. For each microbe, the Shapley value represents the average contribution of that microbe across all possible coalitions. The calculation involves determining how much adding a particular feature to a coalition changes the prediction outcome compared to the coalition without that feature. For each sample in the data set, the SHAP algorithm evaluates the contribution of each microbe to the prediction outcome. It considers subsets of microbes and calculates the difference in prediction outcomes when adding or removing each microbe. The shapely values are then aggregated across all samples in the data set to provide an overall measure of feature importance.

### Differential abundance analysis

Microbial abundance and microbial gene abundance were analyzed using the DESeq2 package (88). DESeq2 uses a median normalization method, which normalizes unequal sampling fractions, ensuring that differences in sequencing depth between samples are minimized in the downstream analysis. Age, gender, and BMI characteristics were included as covariates whenever available. The microbial species abundance data were represented as count matrices, where rows corresponded to microbial species and columns represented samples. Healthy controls were specified as reference. Microbial species were ranked with 5% or 95% confidence intervals (CI) of the LFC depending on whether it decreased or increased in disease cases. Differentially abundant microbes were identified based on the ranking. Microbes with 5% CI of LFC ranked top are termed case-associated microbes, and microbes with 95% CI of LFC ranked bottom are termed control-associated microbes. The analysis of differential gene abundance followed a similar approach as the microbial abundance analysis. Differential gene abundances were generated with the eggNOG annotations (89). The count matrix was created with genes as rows and samples as columns. The LFCs were calculated for further analysis.

### Disease similarity analysis

Disease similarity at the microbial species level was measured using the overlap of differentially abundant microbes. We looked at the top 100 case-associated and top 100 control-associated microbes. Concentrating on these top microbes allows us to prioritize the most relevant and significant microbial species associated with disease status. To investigate the shared microbial signatures between diseases at the species level, pairwise comparisons were conducted to determine the number of overlapping differentially abundant microbes. Similarity networks were plotted with the NetworkX Python package (https://networkx.org/). Diseases within the top pairs that showed high/low similarities (CD, UC, PD, T2D, CRC, schizophrenia, and AD) were included for simplicity. The weight of the edges is proportional to the overlapped differentially abundant microbes in each disease (case vs control), and they are color-coded by case-associated (salmon color) and control-associated (blue color) categories. Disease similarity at the microbial gene level was represented using Pearson’s correlation coefficient (*R*) of LFCs between two diseases. While Spearman correlation analysis is often recommended for microbial count data due to its robustness to non-normality, Pearson correlation analysis can provide a straightforward measure of the strength and direction of the linear relationship between variables. Here, we examined the correlations of LFCs for microbial gene count data across different diseases. Pearson *R* has a clear interpretation of linear association, which may be more intuitive in this situation. A higher positive correlation coefficient value indicates a stronger similarity in the differentially abundant pattern, while a negative correlation value represents how reversed the patterns are. An absolute value of Pearson *R* within the range of 0.5–0.7 would be considered a strong correlation, while ≥0.7 would be considered a very strong correlation.

### Microbial gene analysis

In order to determine if a particular gene is more commonly observed in case-associated microbes or control-associated microbes than by random chance, a genome-wide binomial test (15) was performed between two groups of taxa. Briefly, the gene abundance matrices for each microbe group were used as the input, where rows represent taxa and columns represent genes. For each gene, the null hypothesis posits that the probability of observing it in one group is equal to the probability of observing it in the other group. The significance level for the test was set as 0.001. Microbial genes identified that were statistically significant were subsequently mapped to the KEGG pathways to elucidate their respective functional roles.

## Data Availability

The following publicly available data sets were downloaded through the NCBI SRA using the following accession numbers: PRJEB47976 for Laske et al. (19); PRJNA451479 for Dan et al. (20); ERP104786 for Wang et al. (36); PRJNA686821 for Wan et al. (22); PRJNA400072 for Franzosa et al. (23); PRJEB15371 for a Chinese CD cohort; ERP008729 for Feng et al. (24); ERP005534 for Zeller et al. (25); PRJEB27928 for Wirbel et al. (26); PRJEB32762 for iMSMS Consortium (37); PRJEB4336 for Le Chatelier et al. (28); ERA000116 for Qin et al. (29); PRJNA834801 for Wallen et al. (30); PRJNA433459 for Qian et al. (31); ERP111403 for Zhu et al. (32); PRJNA231909 for Guittar et al. (33); PRJNA422434 for Qin et al. (34); PRJEB1220 for Nielsen et al. (35); PRJNA400072 for Franzosa et al. (23); PRJEB10878 for Yu et al. (38). The pipeline built in this study is available at https://github.com/jindongmin/snakemake_metagenomics. The following softwares and databases were used: Snakemake v 7.1.1, Bracken v2.5.2, Kraken v2.1.2, fasterq-dump v3.0.0, uhgg v2 24 February 2022, q2-sample-classifier v 2022.8.0, pydeseq2 v 0.2.1, and shap v0.41.0.

## SUPPLEMENTAL MATERIAL

Table S1a msystems.00295-24-s0001.txt Shapley values for CD cases.

Table S1b msystems.00295-24-s0002.txt Shapley values for CD controls.

Table S2a msystems.00295-24-s0003.txt Shapley values for CRC cases.

Table S2b msystems.00295-24-s0004.txt Shapley values for CRC controls.

Table S3 msystems.00295-24-s0005.csv *P*-values for the Pearson correlation between diseases.

Fig. S1 msystems.00295-24-s0006.pdf Shared differential abundant microbes.

ASM does not own the copyrights to Supplemental Material that may be linked to, or accessed through, an article. The authors have granted ASM a non-exclusive, world-wide license to publish the Supplemental Material files. Please contact the corresponding author directly for reuse.
