# Supplementary material for: Meta-analysis of the human gut microbiome uncovers shared and distinct microbial signatures between diseases
Source: mSystems. 2024 Jul 30;9(8):e00295-24. doi: 10.1128/msystems.00295-24 (PMC11334437; doi:10.1128/msystems.00295-24)
Supplement: Fig. S1 — Shared differential abundant microbes. [file msystems.00295-24-s0006.pdf]

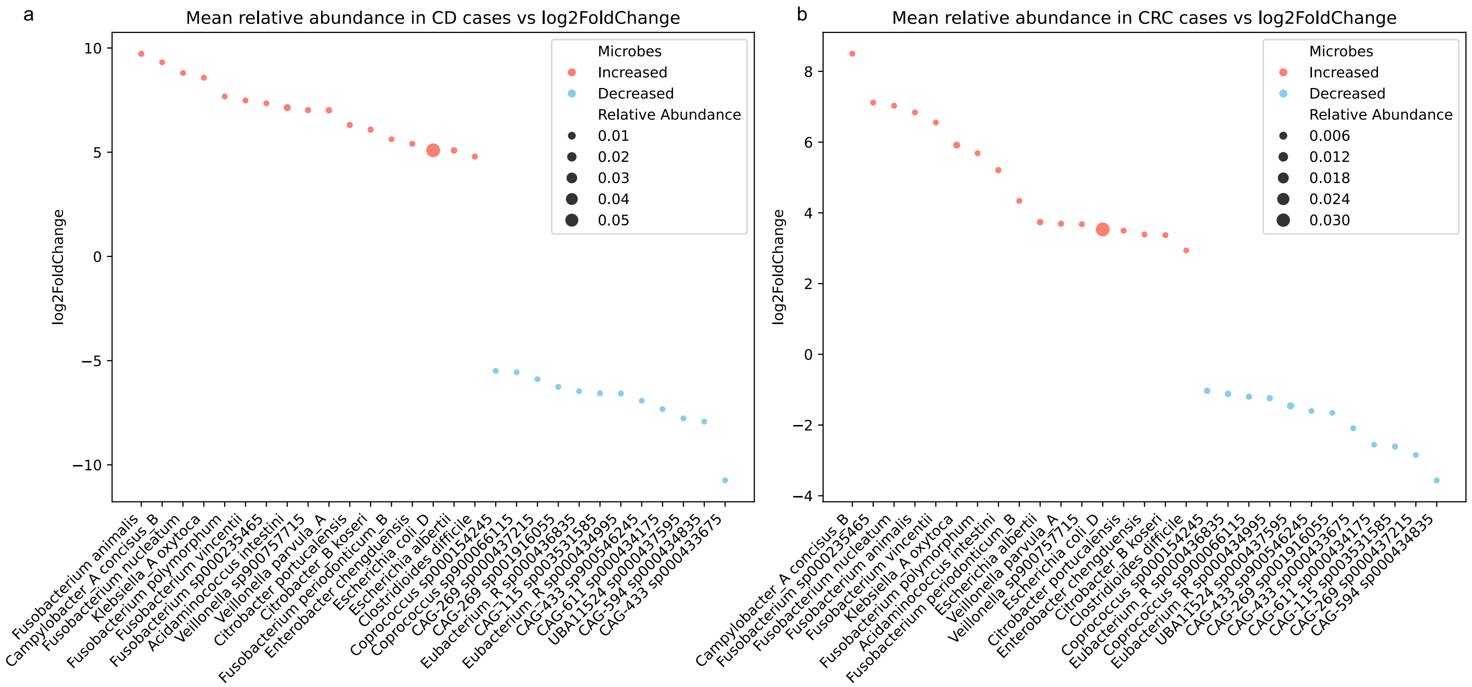

Supplementary Figure 1 Mean relative abundance vs log2FoldChanges for the overlapped differential abun-dant microbes in CD and CRC patients.

The X axis are the shared differential abundant microbes, salmon colored are the ones increased in disease cases, and blue colored are the ones decreased in disease cases. The Y axis are the values of log2FoldChange comparing case and control within each disease. The size of the dots is proportional to the mean relative abundance of each microbe in the disease case group.
